# The effect of teacher self-efficacy, online pedagogical and content knowledge, and emotion regulation on teacher digital burnout: a mediation model

**DOI:** 10.1186/s40359-024-01540-z

**Published:** 2024-01-26

**Authors:** Xianbi Yang, Juan Du

**Affiliations:** 1 Hangzhou Normal University, Hangzhou, 311121 China; 2 ShangHai Baoshan Sparetime University, Baoshan Branch School of ShangHai Open University, ShangHai, 200940 China

**Keywords:** Online teaching competence, Self-efficacy, Digital burnout, Emotion regulation, Chinese teachers

## Abstract

**Background:**

With the increasing prevalence of online teaching, understanding the dynamics that impact educators' well-being and effectiveness is paramount. This study addresses the interconnected relationships among online teaching competence, self-efficacy, emotion regulation, and digital burnout among teachers in the digital learning environment.

**Objectives:**

The primary objectives of this research are to investigate the direct and mediated effects of online teaching competence and self-efficacy on emotion regulation and digital burnout among teachers. Additionally, the study aims to explore the mediating role of emotion regulation in the relationship between self-efficacy and digital burnout. The overarching goal is to provide comprehensive insights into the factors influencing teacher well-being in the online teaching context.

**Methodology:**

A cross-sectional survey design was employed, involving a convenience sample of educators from a specific university. Participants responded to validated self-report measures assessing online teaching competence, self-efficacy, emotion regulation, and digital burnout. Statistical analyses, including regression and mediation analyses, were conducted to examine the relationships among the key variables.

**Results:**

The findings reveal significant relationships and effects among the investigated variables. Online teaching competence is a substantial predictor of emotion regulation and digital burnout. Similarly, self-efficacy significantly impacts emotion regulation and digital burnout. Emotion regulation mediates the relationship between online teaching competence, self-efficacy, and digital burnout. These results highlight the intricate connections shaping teachers' experiences in the digital teaching environment.

**Conclusions and implications:**

In conclusion, this study provides robust evidence supporting the interconnectedness of online teaching competence, self-efficacy, emotion regulation, and digital burnout among teachers. The implications underscore the importance of fostering these competencies through targeted professional development. Educational institutions and policymakers can use these insights to implement strategies that enhance teacher well-being, ultimately promoting a more effective and sustainable online teaching environment.

## Introduction

Distance education has been a noteworthy expansion in recent years, evidenced by the escalating registration rates [1]. This surge underscores the imperative to design a flexible learning environment that caters to the diverse needs of students engaged in online learning. The growth in this sector substantiates the efficacy of the online learning experience in enhancing learners' achievements. Recognizing the significance of students' evaluations of teaching quality and effectiveness is crucial as it is a critical indicator to mitigate a high attrition rate among online learners [1]. Online instructors are encouraged to cultivate proficiency in many skills and abilities essential for effective teaching in technologically integrated settings, ultimately ensuring learner success. Therefore, it is imperative to identify and emphasize teaching behaviors that can adequately inform instructors of the requisite abilities and competencies for positive online teaching.

The online learning setting diverges significantly from the traditional learning context, eliminating the need for physical attendance in classrooms and opportunities for face-to-face interactions with peers and instructors. Online learners are required to be independent, managing their own learning pace [2–5]. Consequently, self-efficacy and proficiency in maximizing online learning technology become pivotal for successful online course completion. Essential skills include the adept use of e-mail, engagement in discussion boards, and proficiency utilizing Internet browsers. Students developing apprehension toward computer technologies may experience confusion, anxiety, and frustration, leading to technology withdrawals. Crucial skills encompass mastery of discussion boards, effective Internet search capabilities, and adept use of e-mail. Indicators manifesting students' fear of computer technology include feelings of confusion, a loss of personal control, anxiety, frustration, and complete disengagement from computer technology [6].

In educational systems, instructors assume a pivotal role as key stakeholders, wielding the influential capacity to impact individual student achievements and the overarching performance of the system [7]. Among the critical teacher variables, burnout is an inability to manage work-related anxiety effectively, strained social relationships, persistent exhaustion, and a waning interest in the teaching profession [8]. Extensively explored in educational research, teacher burnout remains a pervasive concern with substantial implications for teacher well-being, job satisfaction, and student outcomes [9–13]. Recent years have underscored the significance of teacher emotion regulation and self-efficacy as components of teacher well-being and preventive measures against burnout [14]. In teacher training programs, addressing teachers' burnout and stress gains prominence due to their potential contribution to teacher attrition [15, 16].

The concept of self-efficacy, rooted in social cognitive theory, emphasizes human agency's development and utilization, asserting that individuals can exert control over their behavior [17–21]. Within this framework, teacher self-efficacy denotes a teacher's confidence in their ability to organize, plan, and execute activities for achieving specific educational objectives. Demonstrably linked to teacher performance, job satisfaction, and overall well-being, high levels of self-efficacy empower teachers to persist in the face of challenges, fostering effective instructional practices [22–27]. Teacher self-efficacy refers to educators' belief in their ability to successfully perform digital teaching tasks and effectively manage challenges encountered in online education. This variable encompasses teachers' confidence in their technological skills, instructional methods, and overall competence in the digital teaching environment [26].

Moreover, online pedagogical and content knowledge represent teachers' proficiency and expertise in using digital tools for educational purposes. This involves their understanding of effective online teaching strategies, the utilization of relevant technological resources, and the adaptation of content to suit online learning environments. The integration of pedagogical and content knowledge is crucial for teachers to navigate the complexities of online education successfully.

This study also explores emotion regulation as another variable of interest, defined as the ability to control, modify, and manage the awareness and expression of emotions influenced by internal and external factors [27, 28]. Emotion regulation involves individuals' efforts to influence emotional experiences to align with their goals [29]. Emotions, intra-psychological factors, and external elements play a crucial role in the teaching profession, significantly impacting teachers' performance and academic achievements. Recognizing and effectively managing these emotions is paramount for teachers [28]. Consequently, the ability of instructors to regulate and control emotional experiences within the classroom context is termed teacher emotion regulation [30]. This regulation encompasses how teachers perceive, express, modify, maintain, and generate emotional interactions.

Finally, emotion regulation is a psychological variable that pertains to teachers' ability to manage and modulate their emotional responses in the online teaching context. This involves strategies for coping with stress, frustration, or any negative emotions that may arise during the digital teaching experience [27]. Effective emotion regulation is essential for sustaining teacher well-being and preventing burnout in the online teaching environment [28]. The study proposes a mediation model, suggesting that teacher self-efficacy and online pedagogical and content knowledge may influence teacher digital burnout through their impact on emotion regulation. In essence, the model posits that teachers with higher self-efficacy and proficient online pedagogical and content knowledge are better equipped to regulate their emotions, consequently reducing the likelihood of experiencing burnout in the digital teaching domain. Despite the significance of constructs such as teacher self-efficacy, teachers’ teaching competence, emotion regulation, and burnout, there needs to be more research examining the interconnections among these elements, particularly within the context of Chinese high school teachers. Consequently, this study aims to scrutinize the relationships among teachers’ online teaching competence, teacher self-efficacy, emotion regulation, and burnout among Chinese high school teachers.

## Literature review

### Teachers’ competence for online teaching

Numerous competencies are recognized as exemplary practices for online teachers, as indicated by research [31, 32]. However, scrutinizing these identified competencies has uncovered inconsistencies, particularly in models specifically designed for online teaching contexts. Baran et al. [33] noted that the essential roles and competencies expected from online teachers often diverge in the literature, contingent on the context of online teaching. Consequently, the dynamic learning environment demands educators to possess diverse competencies.

In a comprehensive review, Thomas and Graham [34] highlighted that previous research had evaluated various online teaching competencies, with course design emerging as the most extensively studied competency. Conversely, Bigatel et al. [1] outlined online teaching competencies that focused solely on teaching behaviors. They elucidated tasks related to extensive instructional elaboration involving evaluators, course developers, online learning instructors, and academicians, comprising 64 online teaching behaviors. The study engaged 197 respondents, who used a 7-point Likert scale to rate their agreement with each assessment, identifying tasks deemed most critical in online teaching courses. Employing exploratory factor analysis, the study clustered the tasks into seven competency groups: (1) administration/leadership, (2) active learning, (3) multimedia technology, (4) active teaching/responsiveness, (5) technological competence, (6) policy enforcement, and (7) classroom decorum. Bigatel et al. [1] introduced a model elucidating educators' teaching behaviors during course delivery, which did not emphasize the factor of course design. This model serves as the foundation for the current study. Recognizing potential limitations, further validation checks, or suggestions for improvement are essential to assess its accuracy.

## Teachers’ self-efficacy

Bandura [35] defines teacher self-efficacy as the belief in one's ability to accomplish specific tasks successfully. Rooted in Rotter's [36] work and Bandura's [35] social cognitive theory, the concept gained prominence in the late 1970s, mainly through the Rand Corporation's pioneering study. According to Bandura [35], teacher self-efficacy pertains to educators' belief in their abilities to manage specific teaching tasks at a desired level of quality within a given context. This definition underscores individuals as self-organizing, active, self-regulating, and reflective.

In L2 research, self-efficacy has garnered significant attention due to its profound impact on individuals' activity choices, effort investment, and persistence amid challenges [37, 38]. Efficacy beliefs shape perceptions of opportunities and obstacles encountered in language learning, influencing activity choices, effort levels, and perseverance [17, 18].

Teacher self-efficacy is positively associated with work satisfaction, engagement, and organizational commitment, while negatively correlated with burnout [23, 39]. High self-efficacy among instructors correlates with effective collaboration, less inappropriate student behavior, and enhanced ability to achieve shared educational goals [40]. Similar outcomes are observed in studies with large teacher samples, where high self-efficacy is linked to increased work satisfaction and reduced emotional exhaustion [23].

Research suggests that instructors with high self-efficacy contribute to a high-quality learning environment by designing challenging lessons, skillfully managing misbehaviors, and engaging students meaningfully [41]. Recent investigations, such as the study by Sulla and Rollo [42], highlight the positive relationship between teachers' self-efficacy and job satisfaction. Higher self-efficacy levels are associated with increased satisfaction in the teaching profession.

Instructors with higher self-efficacy establish an environment conducive to building stronger bonds with students and engaging in ways supporting students' behavioral functioning [17–20]. Studies with Croatian teachers [43] and EFL practitioners [11] further support the positive impact of teacher self-efficacy on job involvement, satisfaction, and emotional well-being. Emphasizing the influence of culture and context, Hoang and Wyatt [44] underscore the critical role of these factors in shaping self-efficacy beliefs, pedagogical approaches, and classroom management strategies among Vietnamese pre-service teachers.

## Teachers' emotion regulation

Emotion regulation has gained prominence in L2 education, driven by an increasing interest in positive psychology and a desire to understand its impact on L2 teachers and learners [45]. According to Gross [46], emotions arise from repeated patterns of attention and reaction, and their impact can be either constructive, enhancing decision-making skills, or detrimental, leading to maladaptive cognitive or behavioral biases. Emotion regulation involves controlling which emotions individuals feel when they feel and how they express and experience them [46, 47].

This multifaceted concept has been described in various ways. Gross [47] defines emotion regulation as the process by which individuals control their emotions, encompassing the selection, modification, and expression of emotions. Thompson et al. 34) expand this definition, including internal and external processes that involve evaluating and managing emotions to achieve personal objectives. Cole et al. [48] conceptualized emotion regulation as the ability to react to life's circumstances with a range of emotions in a socially acceptable and adaptable manner, allowing for spontaneous and delayed reactions as appropriate.

Emotion regulation can be categorized into downregulation and upregulation [49]. Downregulation aims to minimize and regulate the impacts of negative emotions, while upregulation seeks to enhance and amplify positive emotions. Instructors often employ emotion regulation strategies in the teaching profession, where interactions between teachers and students are prevalent. Downregulation can mitigate negative emotions, such as stress, that may impede students' motivation, engagement, and achievement [45]. Conversely, upregulation techniques can enhance teaching effectiveness and promote academic accomplishment [50].

Gross [47] distinguishes between intrinsic and extrinsic emotion regulation. Intrinsic emotion regulation occurs when an individual regulates their emotions, while outside emotion regulation involves attempts to control another person's feelings. Teachers play a crucial role in language classrooms in helping learners manage their emotions through external support, especially in emotionally vulnerable situations during learning [51].

Despite a growing interest in emotion regulation across various disciplines, limited research has focused on language education, particularly language teachers' emotion regulation [51, 52]. In classes, both instructors' and pupils' positive and negative emotions influence learning outcomes, with positive emotions enhancing learning and negative emotions hindering it [49]. Various studies in different cultural settings have explored teachers' emotion regulation strategies and their impact on professional goals and overall well-being [53–58].

## Teachers’ digital burnout

The concept of "burnout" was first introduced by Freudenberger [55] to describe a psychological condition resulting from continuous workplace stressors. According to Maslach and Jackson [56], burnout encompasses three dimensions: emotional exhaustion, depersonalization, and reduced personal accomplishment. Emotional exhaustion reflects a person's diminished emotional state due to stress and work overload, while depersonalization involves negative attitudes toward students or coworkers. A negative self-perception and a sense of ineffectiveness characterize reduced personal accomplishment. Long-term job stress, especially in human service professions like teaching, is considered a primary cause of burnout [57].

People in human service fields, including education, frequently experience burnout [58]. Teachers often experience exhaustion due to job demands and other responsibilities [59]. The educational environment, including student misbehavior, work-related stress, lack of support, interpersonal issues, and role ambiguity, is a primary cause of burnout among teachers [60]. Teachers' psychological inclinations, particularly self-efficacy beliefs, are crucial in coping with these stressors [61]. Recent investigations have also explored the interplay between teachers' emotions, technostress, and burnout in the context of distance learning during the pandemic [62].

Technology usage, such as work, communication, online shopping, social media, and news consumption, has surged during the pandemic. Sharma et al. (2020) note that individuals spent much of their waking hours online during COVID-19 quarantine. However, this heavy reliance on technology, both in professional and social spheres, has resulted in negative consequences, including stress, fatigue, decreased performance, and burnout. Burnout, defined by the World Health Organization in 2019, is a professional deformation affecting individuals' health [63].

Burnout is characterized by a loss of power and a lack of effort. Maslach and Jackson [56] describe it as emotional burnout and desensitization syndrome, often arising from working with people. Pines and Aronson [64] view burnout as a physical, emotional, and mental breakdown, leading to a loss of capacity, energy, idealism, and purpose, accompanied by feelings of pessimism, despair, and entrapment. Studies suggest that burnout is common among professions involving continuous interaction with people, such as teaching [56, 65]. Teachers, in particular, may experience burnout due to factors like student discipline problems, overcrowded classrooms, communication challenges with parents, professional dissatisfaction, unfair administrators, and low income [66]. Burnout can manifest in various ways, encompassing physical and psychological aspects [67]. In the current informative era, a new form of burnout, digital burnout, has become more prevalent.

The COVID-19 pandemic has compelled teachers to rely heavily on technology, especially with the shift to distance education. Teachers have immersed themselves in digital tools, extending their traditional working hours to adapt to the new distance education system. This constant exposure to digital tools 24/7 has given rise to digital burnout, characterized by stress, fatigue, desensitization, decreased attention, and physical and mental health issues [68].

## Theoretical framework

In line with the related literature, the following theoretical framework (Hypothetical Model of the relationship between the variables) is stated.

## Research hypotheses

In line with the existing gap and objectives of the study, the following hypotheses are stated.

H1 = Teachers' online teaching competence has a significant effect on emotion regulation.

H2 = Teachers' online teaching competence has a significant effect on digital burnout.

H3 = teachers’ emotion regulation mediates the effect of teachers’ online teaching competence on digital burnout.

H4 = teachers' self-efficacy affects teachers' emotion regulation.

H5 = teachers' self-efficacy affects teachers' digital burnout.

H6 = teachers’ emotion regulation mediates the effect of teachers’ self-efficacy on digital burnout.

H7 = Teachers' emotion regulation affects digital burnout.

## Methodology

This section provides an in-depth overview of the methodology employed in the research study, focusing on the participants, instruments used for data collection, the procedure followed, and the subsequent data analysis approach.

## Participants

The participants in this study were 450 full-time and part-time lecturers from Hangzhou Normal University who voluntarily responded to the questionnaires. The participants were all selected through convenience sampling. The required sample size for structural equation modeling is 384, however to be on the safer ground, we called out for a larger number of participants. The initial call-out involved 450 lecturers, but only 390 individuals returned the completed questionnaires. Participants were selected using convenience sampling. Among the respondents, 60% held a Ph.D., while 40% were either Ph.D. candidates or held a Master's degree, and they served in roles such as teacher assistants or part-time teachers. The gender distribution of the participants was 63% male and 37% female. Participants spanned an age range of 29 to 60 years, with a mean age (M) of 41 years and a standard deviation (SD) of 5.25. The distribution of participants based on teaching experience revealed that 30% had more than 2c0 years of teaching experience, 40% had between 10 and 20 years of experience, and the remaining 30% had less than ten years of teaching experience.

## Instruments

In this study, four instruments were utilized to assess various aspects.

### a. Online teaching competence scale

The first instrument, the Online Teaching Competence scale, is a 30-item scale employed to evaluate online teaching competencies [1]. Sample items included assessing instructors' encouragement of student interaction through team tasks and projects and monitoring adherence to academic integrity policies. The scale demonstrated good internal consistency, with a calculated Cronbach's alpha of 0.83.

### b. Teacher self-efficacy scale (TSES)

The second instrument, the Teacher Self-Efficacy Scale (TSES), is a 12-item self-report scale designed to assess teachers' beliefs in their ability to achieve desired instructional outcomes [22]. Participants rated their agreement on a 7-point Likert scale, and the reliability of the scale was found to be within an acceptable range, as reported in the results section. The instrument had a good internal consistency, with a calculated Cronbach's alpha of 0.85.

### c. Emotion regulation questionnaire (ERQ)

The third instrument used in the study is the Emotion Regulation Questionnaire (ERQ), a 10-item self-report scale developed by Gross and John [46]. It evaluates individuals' capacity to regulate emotions through cognitive reappraisal and expressive suppression. Participants rated their agreement on a 7-point Likert scale. The scale's validity and reliability were substantiated in by Li [69], providing empirical support for its efficacy in measuring emotion regulation accurately. The reliability of this scale for this study was obtained to be 0.88.

### d. Digital burnout scale

The fourth instrument used for data collection in this study was the "Digital Burnout Scale," developed by Erten and Özdemir [68]. This scale was designed to gauge individuals' levels of digital burnout and includes three sub-dimensions: "digital aging," "digital deprivation," and "emotional exhaustion." The researchers established the validity of this scale in terms of item content and construct validity. Additionally, the Cronbach Alpha coefficient for the scales was calculated as above 0.82 for each scale and its components, indicating an acceptable level.

## Procedure

The study commenced by securing ethical approval from the Institutional Review Board, a crucial step aimed at safeguarding the rights and welfare of the participants. The comprehensive research protocol, encompassing data collection procedures and the informed consent process, underwent a meticulous review and received formal approval. Chinese online platforms were deliberately chosen for data collection to align with approved ethical guidelines.

Prior to the survey initiation, participants were provided with a detailed explanation elucidating the study's purpose and procedures through emails. They were explicitly made aware of the voluntary nature of their participation, the assurance of anonymity in their responses, and the commitment to handle their data with the utmost confidentiality. Participants were reassured that their decision to participate or withdraw would have no bearing on their professional standing or their relationship with the institutions involved in the study. The survey questionnaire consisted of two main parts: the first segment focused on gathering demographic information, while the second part incorporated four validated self-report measures assessing the targeted constructs. The assessment utilized a Likert scale format, providing a structured framework for participants to express their opinions. Clear instructions were given, urging participants to complete the survey independently and allocate sufficient time for thoughtful and accurate responses.

The data collection phase extended over a period of two months, spanning from June to July 2023. During this timeframe, the online survey was administered, ensuring that data collection occurred within the designated window. This meticulous approach to data collection further underscores the study's commitment to adhering to ethical standards and ensuring the integrity of the research process.

## Data analysis

Data analysis involved descriptive and correlation analyses using SPSS 23.0. The study's hypothesis was tested through Structural Equation Modeling (SEM) in the Amos program (version 22.0), as the data were all normalized. Initially, the measurement model was fitted to the data, followed by examining the underlying structural model. Fit indices such as χ2/df, GFI, CFI, RMSEA, and SRMR were employed to assess the overall fitness of the hypothesized model. A χ2/df of less than 3 with a p-value more significant than 0.05 was considered good, and GFI and CFI values of 0.90 or higher indicated a good fit. RMSEA < 0.08 and SRMR < 0.10 were considered favorable fit indices [70].

## Results

Prior to commencing the analysis, a series of checks were conducted to address potential issues with missing data, normality, and outliers. The examination of missing data revealed that less than 2% of the dataset was missing, and to handle this, the expectation–maximization algorithm (EM) was employed. To assess the normality assumption, skewness and kurtosis values were examined, both of which were found to fall within the acceptable range of ± 2.0. This confirmed that the data distribution met the criteria for normality.

Identification of univariate and multivariate outliers was performed using Mahalanobis distance. The analysis detected three cases as multivariate outliers, and as a result, these instances were subsequently excluded from the subsequent analysis to ensure the robustness and validity of the findings.

Table 1 presents the outcomes of descriptive and correlation analyses conducted on the various constructs. The table illustrates a notable positive correlation between the competence of teachers in online teaching and both emotion regulation (*r* = 0.60, *p* < 0.01, df = 388) and self-efficacy (*r* = 0.70, *p* < 0.01, df = 388). This implies that elevated levels of online teaching competence among teachers are associated with increased levels of emotion regulation and self-efficacy. Furthermore, a significant positive correlation is observed between emotion regulation and teacher self-efficacy (*r* = 0.57, *p* < 0.01, df = 388), indicating that heightened emotion regulation is connected to enhanced self-efficacy among teachers. Additionally, teacher digital burnout demonstrates a significant negative correlation with teacher online teaching competence (*r* =  − 0.55, *p* < 0.01, df = 388), emotion regulation (*r* =  − 0.60, *p* < 0.01, df = 388), and teacher self-efficacy (*r* =  − 0.56, *p* < 0.01, df = 388). This suggests that increased levels of teacher burnout are linked to decreased levels of online teaching competence, emotion regulation, and self-efficacy among teachers.

**Table 1:** Descriptive statistics of the variables

	M	SD	1	2	3	4
Teaching competence	3.56	0.64	1			
Self- efficacy	4.25	0.56	0.65	1		
Emotion regulation	5.23	1.1	0.60	0.57	1	
Digital burnout	2.7	0.53	-0.55	-0.56	-0.60	1

## Results of confirmatory factor analysis (CFA)

CFA was employed to evaluate the measurement model, revealing that the four-factor model encompassing teacher self-efficacy, teacher resilience, emotion regulation, and teacher burnout exhibited a satisfactory fit to the data (χ2/pdf = 1.87, CFI = 0.93, TLI = 0.92, RMSEA = 0.06, SRMR = 0.04). Detailed results of the confirmatory factor analysis, including factor loadings, standard errors, and fit indices for each scale, are presented in Table 2.

**Table 2:** Confirmatory factor analysis (CFA)

Scale	Components	Factor 1	Factor 2	Factor 3	Factor 4
Teacher self-efficacy	TE1	0.82			
	TE2	0.83			
	TE3	0.78			
Online teaching competence	OT1		0.78		
	OT2		0.79		
	OT3		0.80		
	OT4		0.83		
	OT5		0.86		
Emotion regulation	ER1			0.76	
	ER2			0.79	
	ER3			0.83	
Digital burnout	DB1				0.86
	DB2				0.83
	DB3				0.81

## Convergent validity and reliability of the constructs

Convergent validity and composite reliability of the constructs are presented in Table 3. An AVE score equal to or exceeding 0.6 indicates that a minimum of 60% of the variability in the construct is accounted for by its indicators, signifying robust convergent validity. Additionally, a CR score of 0.7 or higher represents strong reliability. As per the table, all constructs exhibit commendable convergent validity, given that their AVE values surpass 0.6. Furthermore, each construct demonstrates sound internal consistency, with CR values exceeding 0.80, implying that the items within each construct reliably measure the same underlying concept.

**Table 3:** Convergent validity and composite reliability

Constructs	AVE	Composite reliability
Teacher self-efficacy	0.62	0.86
Online teaching competence	0.63	0.90
Digital burnout	0.55	0.86
Emotion regulation	0.64	0.88

## Divergent validity of the constructs

The divergent validity of the constructs is presented in Table 4.

**Table 4:** Divergent validity of the variables

	**1**	**2**	**3**	**4**
Teacher self-efficacy	0.80			
Online teaching competence	0.56	0.78		
Digital burnout	0.51	0.53	0.80	
Emotion regulation	0.45	0.54	0.60	0.83

Furthermore, as indicated in Table 4, the diagonal elements showcase the square root of AVE for each construct, with values of 0.80, 0.78, 0.81, 0.83, and 0.85 for teacher self-efficacy, teacher online teaching competence, teacher burnout, digital burnout, and emotion regulation respectively. These figures serve as a measure of the shared variance among the indicators within each construct. On the other hand, the off-diagonal elements depict the correlation coefficients between the constructs. Notably, all off-diagonal elements exhibit values lower than the corresponding diagonal elements, providing empirical evidence for the discriminant validity of the constructs. This implies that each construct is distinct, supporting the idea that the measurement model accurately distinguishes between the different latent variables.

Several structural models were examined after validating the measurement model to assess the hypotheses. The study compared three models: the hypothesized partial mediation model (Model 3), a full mediation model (Model 2), and a direct model (Model 1). The fit statistics for each model are presented in Table 6. The findings indicated that Model 3 exhibited a significantly better fit than both Model 2 (Δdf = 6, Δχ2 = 85.57, *p* < 0.001) and Model 1 (Δdf = 5, Δχ2 = 259.33, *p* < 0.001), as evidenced by the utilized fit indices. Consequently, Model 3 was considered the most economical fit for the data. Results are shown in Table 5.

**Table 5:** Comparison of fit indices for three models

	X^2^	Δχ2	CFI	RMSEA	TLI	SRMR	GFI
Direct effect (1)	1049		0.95	0.05	0.92	0.16	0.84
Full mediation (2)	750	260	0.91	0.03	0.94	0.06	0.86
Partial mediation (3)	706	851	0.97	0.04	0.95	0.07	0.87

**Fig. 1:**
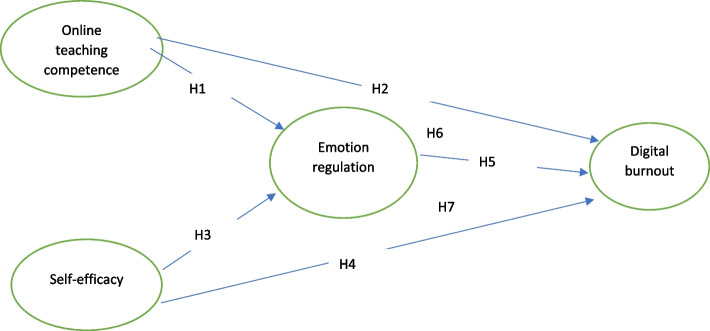
Hypothetical model of the relationship between the variables

The final fitted model (Partial Mediation) is depicted in Fig. 1, illustrating the path and parameter estimates. Notably, path coefficients were significant for all paths except the one linking teacher emotion regulation and burnout. The finalized fit model (Partial Mediation) is depicted in Fig. 1, showcasing the path and parameter estimates. All path coefficients were found to be statistically significant. Subsequently, Baron and Kenny's (1986) approach was employed by the researcher to investigate whether teacher resilience acted as a mediator in the relationship between variables. The direct model (Table 6) revealed significant path coefficients between the constructs. Results are shown in Table 6.

**Table 6:** Path estimates of the structural model

Results	Effect sizes			Research hypotheses
	Standard Coefficients	Non-standard coefficients		
		***p***	**T**	
accepted	0.778	*P* < 0/01	20.070	H1 = Teachers' online teaching competence has a significant effect on emotion regulation
accepted	0.45	*P* < 0/01	10.23	H2 = Teachers' online teaching competence has a significant effect on digital burnout
accepted	0.70	*P* < 0/05	14.25	H3 = teachers’ emotion regulation mediates the effect of teachers’ online teaching competence on digital burnout
Accepted	0.67	*P* < 0.01	7.43	H4 teachers' self-efficacy affects teachers' emotion regulation
accepted	0.77	*P* < 0/01	21.20	H5 teachers' self-efficacy affects teachers' digital burnout
Accepted	0.53	*P* < 0/01	9.92	H6 teachers’ emotion regulation mediates the effect of teachers’ self-efficacy on digital burnout
Accepted	0.413	*P* < 0/01	9.23	H7 = Teachers' emotion regulation affects digital burnout

The study's findings reveal significant effects and relationships among key variables, providing valuable insights into the dynamics of online teaching competence, emotion regulation, self-efficacy, and digital burnout among teachers. Firstly, the analysis supports Hypothesis 1, indicating that teachers' online teaching competence has a substantial impact on emotion regulation (*p* < 0.01, T = 20.070). This suggests that the proficiency of teachers in online teaching significantly influences their ability to regulate emotions in the digital teaching environment. Furthermore, Hypothesis 2 is confirmed, showing a significant effect of teachers' online teaching competence on digital burnout (*p* < 0.01, T = 10.23). This highlights the critical role of online teaching competence in mitigating or exacerbating digital burnout among educators. Hypothesis 3 is accepted, indicating that teachers' emotion regulation acts as a mediator in the relationship between their online teaching competence and digital burnout (*p* < 0.05, T = 14.25). This implies that the impact of online teaching competence on digital burnout is, in part, mediated by teachers' ability to regulate their emotions. Moreover, the study supports Hypothesis 4, revealing a significant effect of teachers' self-efficacy on emotion regulation (*p* < 0.01, T = 7.43). This underscores the role of teachers' self-perceived efficacy in influencing their ability to regulate emotions, a crucial aspect of effective teaching. Additionally, Hypothesis 5 is accepted, demonstrating a significant effect of teachers' self-efficacy on digital burnout (*p* < 0.01, T = 21.20). This suggests that teachers' confidence in their abilities significantly impacts the level of digital burnout they experience. Furthermore, hypothesis 6 is supported, indicating that teachers' emotion regulation mediates the relationship between self-efficacy and digital burnout (*p* < 0.01, T = 9.92). This implies that the influence of self-efficacy on digital burnout is, at least partially, mediated by teachers' emotional regulation. Lastly, Hypothesis 7 is accepted, revealing a significant effect of teachers' emotion regulation on digital burnout (*p* < 0.01, T = 9.23). This underscores the importance of emotion regulation as a key factor influencing the occurrence of digital burnout among teachers. In summary, the study provides robust evidence supporting the interconnectedness of online teaching competence, self-efficacy, emotion regulation, and digital burnout among teachers, shedding light on crucial factors that impact their well-being and effectiveness in the digital teaching environment.

## Discussion

The present study builds upon the established literature that underscores the pivotal role of online teaching competence in shaping educators' ability to regulate emotions effectively in the digital teaching environment [1, 32, 33]. Our findings, supporting Hypothesis 1, align with the work of Bigatel et al., which emphasizes the significance of fostering online teaching competence to enhance teacher emotion regulation [1]. This connection is also supported by Gurley's research, which highlights the importance of educators' preparation in influencing their perceived teaching presence, a component closely related to online teaching competence [32].

Our results affirm Hypothesis 1, revealing a substantial positive impact of teachers' online teaching competence on emotion regulation. This aligns with existing literature, suggesting that educators proficient in online pedagogy are better equipped to navigate the challenges of digital teaching, leading to enhanced emotional regulation. Teachers with heightened competence in online pedagogy may experience increased confidence and capability, positively influencing their emotional responses to the demands of digital teaching environments [1, 21].

Moreover, the statistical analysis supports Hypothesis 2, demonstrating a significant negative effect of teachers' online teaching competence on digital burnout. This suggests that as teachers' competence in online teaching increases, the likelihood of experiencing digital burnout decreases. Educators proficient in utilizing digital tools and platforms may find their work more manageable and less overwhelming, contributing to a reduced risk of burnout in the digital teaching context [1, 32]. This finding aligns with the research by Yu et al., which emphasizes the relationship between interaction, internet self-efficacy, and student satisfaction in online education courses [2]. It further resonates with Thompson et al.'s work on successful online teaching, underlining the importance of effective online teaching practices [33].

In addition, the findings robustly support Hypothesis 3, indicating that teachers’ emotion regulation plays a crucial mediating role in the relationship between online teaching competence and digital burnout. This implies that the positive impact of online teaching competence on reducing digital burnout is, in part, facilitated by teachers' effective regulation of their emotions [30, 71]. This aligns with Gross's work, which emphasizes the crucial role of emotion regulation in various contexts [47]. The findings also parallel the research of Wang et al., exploring emotion regulation strategies in second language learning contexts [45].

Furthermore, our study validates hypothesis 4, emphasizing a significant positive effect of teachers' self-efficacy on their emotion regulation. This implies that teachers who possess a strong belief in their ability to perform effectively in their role exhibit better emotional regulation. High self-efficacy might empower educators to approach challenges with confidence, influencing how they interpret and manage their emotions in the teaching context [22, 30, 31, 37]. Similarly, hypothesis 5 is substantiated by the findings, revealing a significant negative effect of teachers' self-efficacy on digital burnout. Higher levels of self-efficacy among teachers are associated with a reduced likelihood of experiencing digital burnout. Educators with strong self-efficacy may perceive challenges as more manageable and may persist in the face of difficulties, contributing to a lower risk of burnout in the digital teaching domain [22, 30].

The statistical analysis also supports hypothesis 6, indicating that teachers’ emotion regulation acts as a mediator in the relationship between self-efficacy and digital burnout. This suggests that the positive impact of self-efficacy on reducing digital burnout is, at least partially, explained by teachers' effective regulation of their emotions. High self-efficacy, coupled with effective emotion regulation, creates a more resilient mindset that helps mitigate the adverse effects of digital burnout [30, 71]. Finally, hypothesis 7 is supported by the results, indicating a significant negative effect of teachers' emotion regulation on digital burnout. Educators with better emotion regulation skills are less likely to experience digital burnout. Effective emotion regulation acts as a protective factor, allowing teachers to cope more adaptively with the challenges posed by digital teaching, thereby reducing the risk of burnout [30–50, 71]. In conclusion, the study's findings provide comprehensive insights into the interplay between teachers' online teaching competence, self-efficacy, emotion regulation, and digital burnout. The identified relationships underscore the importance of addressing both technical and psychological aspects to enhance the well-being and effectiveness of teachers in the digital teaching landscape. These results contribute valuable knowledge to the field and have practical implications for teacher training programs and support initiatives aimed at fostering resilience and competence in the digital education era.

In sum, the results of this study revealed a direct association between teacher burnout and teacher self-efficacy. This discovery aligns with prior research emphasizing the crucial role of teachers' self-efficacy in enhancing their work engagement and enthusiasm [17, 20, 23]. Existing literature has demonstrated that perceptions of self-efficacy are linked to burnout [23]. This sense of assurance could shield teachers from burnout by enabling them to manage the demands of their profession better. Conversely, instructors with lower self-efficacy may be overwhelmed by job demands, potentially increasing their susceptibility to burnout. Therefore, instructors' perceptions regarding their abilities to employ effective teaching methods, manage classroom dynamics, and engage students can significantly impact their susceptibility to burnout [72].

This study's findings align with the conclusions of Skaalvik and Skaalvik [73], who identified a positive connection between job satisfaction and teacher self-efficacy while noting negative correlations with both facets of teacher burnout, with emotional exhaustion emerging as the most influential predictor. Another plausible explanation for this result is that when instructors possess confidence in their abilities and pedagogical competence, they invest more time and effort in their profession, exhibiting more tremendous enthusiasm and experiencing lower levels of burnout.

Teachers proficient in emotion regulation may be better equipped to navigate the challenges inherent in their profession, thereby reducing the likelihood of experiencing burnout [74, 75]. The noteworthy finding that resilience mediates the association between emotion regulation and burnout implies that interventions targeting enhancing emotion regulation skills could effectively mitigate burnout by bolstering teachers' resilience. The partial mediation model indicated that teacher emotion regulation partially mediated the link between teacher self-efficacy, teaching competence, and burnout. Additionally, teacher emotion regulation had an insignificant path coefficient on burnout. Thus, the influence of emotion regulation on teacher burnout played a crucial role in impacting burnout.

## Conclusions and implications

In conclusion, this study illuminates crucial insights into the intricate interplay of online teaching competence, self-efficacy, emotion regulation, and digital burnout among educators. The research findings provide robust evidence supporting the direct and mediated relationships among these key variables. Specifically, teachers' online teaching competence emerged as a significant predictor of emotion regulation and digital burnout. Moreover, self-efficacy demonstrated a profound impact on both emotion regulation and digital burnout. The mediating role of emotion regulation in the relationship between online teaching competence, self-efficacy, and digital burnout underscores the nuanced dynamics in the digital teaching environment. Additionally, the study emphasizes the pivotal role of emotion regulation as a standalone factor influencing digital burnout among teachers.

The implications of these findings are multifaceted. Firstly, institutions and educational policymakers should recognize the importance of cultivating and assessing teachers' online teaching competence, self-efficacy, and emotion regulation skills. Professional development programs should be designed to enhance these competencies, ultimately contributing to educators' well-being and effectiveness in online teaching contexts. Additionally, interventions targeting emotion regulation may be a preventive measure against digital burnout, promoting a healthier and more sustainable teaching environment. Furthermore, the study underscores the need for a holistic approach to teacher well-being, considering the interconnectedness of various factors. Institutions can establish support mechanisms, such as counseling services and stress management workshops, to help teachers cope with the demands of online teaching. Recognizing the impact of emotion regulation on burnout, strategies for fostering emotional well-being should be incorporated into teacher training programs.

## Limitations and suggestions for further studies

Despite the valuable insights provided by this study, it is essential to acknowledge certain limitations. Firstly, using convenience sampling from a specific university may limit the generalizability of the findings to a broader population. Additionally, the reliance on self-reported measures introduces the potential for response bias. The study's cross-sectional nature impedes the establishment of causal relationships among variables. Longitudinal studies offer a more comprehensive understanding of the dynamic interactions over time.

To build upon this research, future studies could employ more diverse samples, encompassing educators from various institutions and cultural backgrounds. Longitudinal research designs could explore the temporal dynamics of online teaching competence, self-efficacy, emotion regulation, and burnout. Qualitative approaches, such as interviews and focus groups, may provide deeper insights into teachers' subjective experiences in the digital teaching environment. Additionally, investigating the efficacy of specific interventions aimed at enhancing emotion regulation and reducing digital burnout could offer practical strategies for educational institutions.

## Data Availability

No datasets were generated or analysed during the current study.
